# Enzymatic aspects in ENT cancer-Matrix metalloproteinases

**Published:** 2014-09-25

**Authors:** AA Zamfir Chiru, CR Popescu, DC Gheorghe

**Affiliations:** * "Grigore Alexandrescu" Hospital, Bucharest, Romania; ** "Colțea" Hospital, Bucharest, Romania; *** "MS Curie" Hospital, Bucharest, Romania

**Keywords:** enzymatic aspects, ENT cancer, matrix metalloproteinases

## Abstract

Abstract

The study of ENT cancer allows the implementation of molecular biology methods in diagnosis, predicting the evolution of the disease and suggesting a certain treatment.

MMPs are proteolytic enzymes, zinc dependent endopeptidases, secreted by tissues and proinflammatory cells that play a role in the clearance of cell surface receptors. They are expressed as zymogens (inactive forms). Proteolytic enzymes cleave zymogens generating active forms.

They are involved in cell proliferation, adhesion, differentiation, migration, angiogenesis, apoptosis and host defense.

Matrix metalloproteinases (MMPs) are proteolytic enzymes that contribute to tissue growth and remodeling and also to tumor expansion by degrading components of the extracellular matrix (collagens, elastins, gelatins, matrix glicoproteins) [**1**]. There are studies about 26 human MMPs, named according to their structure: 

- collagenases (MMP1, MMP13), 

 - gelatinases (MMP2-MMP9), 

 - stromelysins – degrade collagen type IV, but not type I

 - matrilysins (MMP7)

 - membrane-type I - located at an intercellular and intracellular level at the same time. 

 Considering their connection with cell membrane, MMPs are classified into 8 types: transmembrane type I and II, (contains an intracellular or transmembranar domain), gelatin-binding, simple hemopexin, furin activated, vitronectin-like, minimal domain and GPI-linked [**2**].

 MMPs expression and activity is determined by in situ zymography [**3**], gelatin zymography and Western Immunoblot analysis [**4**].

 Extracellular proteolysis is a complex process that involves the degradation of the extracellular matrix and the weakening of intracellular connections, but also has a role in the synthesis of bioactive molecules [**5**].

 MMPs activity is modulated by tissue inhibitors of metalloproteinases (TIMP) [**6**] and also regulated by hormones, growth factor, cytokines.

 In a pathophysiological way, the regulation of pericellular proteolysis contributes to the clinical aspects of the diseases, with both diagnostic and therapeutic significance [**6**].

 Reports revealed a direct involvement of matrix metalloproteinase (MMPs) overexpression in development and progression of ENT squamous cell carcinoma [**7**].

 A correlation between MMP9 expression and angiogenesis has been documented [**8**], p53 status and activity of the iNOS pathway in ENT squamous cell carcinoma. Imbalance between MMP and TIMP is a prognostic indicator of the pathological process, tumor aggressiveness and cancer progression [9,10].

 Cancer progression can also involve focal adhesion kinase (FAK) [**11**]. Overexpression of this kinase has demonstrated to be an early event in squamous cell carcinoma and associated with metastases in cervical lymph nodes. FAK increases cell motility and MMP2 production. The level of expression of MMPs is useful for treatment planning and prognosis, being related to clinical/histological features of the tumor and lymph nodes [**12**].

Expression of MMPs was found in human ENT cancers and was correlated with advanced stage, invasive disease and metastatic properties.

 By remodeling extracellular matrix and releasing growth factors, MMPs provide a favorable environment for the primary tumor.

 In cell survival and proliferation, MMPs play an important role by augmenting "survival signals" [**13**]. The in vivo interaction of MMPs with cell surface receptors and stimulating or inhibiting apoptosis has been demonstrated. MMP2 and MMP9 increase apoptosis during tissue remodeling and neoangiogenesis.

 In cancer, cell apoptosis is decreased because of the bioavailability of VEGF (that is involved in tumor growth and angiogenesis).

 Angiogenic factors (VEGF, βFGF) are secreted by inflammatory or tumor cells. They bind on the surface of the endothelial cells that begin to secret MMPs, change their expression of integrins and undergo proliferation [**14**]. TGFβ induce the secretion of MMPs by endothelial cells and is also a good chemotactic for inflammatory cells and recruits pericytes for the new vessels, to complete their maturation.

 MMPs in extracellular matrix release proangiogenic factors (β FGF,VEGF, TGFβ) [**15**].

 Also, MMPs facilitate endothelial cell migration by removing adhesion sites and cleaving cell-cell and cell-matrix receptors [**10**].

 In conclusion, MMPs are involved in tumoral processes by allowing the tumor cells invade the surrounding stroma (gelatinases, MMP2/MMP9), breaking blood or lymphatic vessels’ basal membrane, generating intravasation and extravasation. They also initiate and sustain the growth of tumor cells (by angiogenesis to metastatic site). MMPs activate mutation of proto-oncogenes k-ras/H-ras in tumor cells (up regulate expression of VEGF and inhibit trombospondine) and erb B2 (stimulate the proangiogenic factor and inhibit trombospondine).

 MMPs inhibitors can be natural or synthetic. They have been documented as cytostatic and antiangiogenic agents. Unfortunately, they have short half-life in vitro.

 MMPs inhibitors were developed from a peptide sequence recognized by the targeted protease that interacts with the zinc of the active site. Synthetic MMP inhibitors are, for example: marimastat, batimastat. They are low molecular weight inhibitors. Natural MMP inhibitors are tetracyclines, neovastat, nicotiamide, rifampicin.

 The development of MMPs inhibitors was a way to improve the selectivity of newly developed drugs toward a certain MMPs, optimizing the interaction with MMPs in ENT cancer patients. An important step in cancer treatment was to understand the mechanism by which drugs elicit their antitumoral and antimetastatic effect.
